# Histological and MRI brain atlas of the common shrew, *Sorex araneus*, with brain region-specific gene expression profiles

**DOI:** 10.3389/fnana.2023.1168523

**Published:** 2023-05-03

**Authors:** Cecilia Baldoni, William R. Thomas, Dominik von Elverfeldt, Marco Reisert, Javier Làzaro, Marion Muturi, Liliana M. Dávalos, John D. Nieland, Dina K. N. Dechmann

**Affiliations:** ^1^Department of Migration, Max Planck Institute of Animal Behavior, Radolfzell am Bodensee, Germany; ^2^Department of Biology, University of Konstanz, Konstanz, Germany; ^3^International Max Planck Research School for Quantitative Behaviour Ecology and Evolution, Konstanz, Germany; ^4^Department of Ecology and Evolution, Stony Brook University, Stony Brook, NY, United States; ^5^Division of Medical Physics, Department of Diagnostic and Interventional Radiology, University Medical Center Freiburg, Faculty of Medicine, University of Freiburg, Freiburg im Breisgau, Germany; ^6^Javier Lázaro Scientific and Wildlife Illustration, Noasca, Italy; ^7^Consortium for Inter-Disciplinary Environmental Research, Stony Brook University, Stony Brook, NY, United States; ^8^Department of Health Science and Technology, Aalborg University, Aalborg, Denmark

**Keywords:** Dehnel’s phenomenon, neocortex, hippocampus, hypothalamus, olfactory bulb, Soricidae

## Abstract

The common shrew, *Sorex araneus*, is a small mammal of growing interest in neuroscience research, as it exhibits dramatic and reversible seasonal changes in individual brain size and organization (a process known as Dehnel’s phenomenon). Despite decades of studies on this system, the mechanisms behind the structural changes during Dehnel’s phenomenon are not yet understood. To resolve these questions and foster research on this unique species, we present the first combined histological, magnetic resonance imaging (MRI), and transcriptomic atlas of the common shrew brain. Our integrated morphometric brain atlas provides easily obtainable and comparable anatomic structures, while transcriptomic mapping identified distinct expression profiles across most brain regions. These results suggest that high-resolution morphological and genetic research is pivotal for elucidating the mechanisms underlying Dehnel’s phenomenon while providing a communal resource for continued research on a model of natural mammalian regeneration. Morphometric and NCBI Sequencing Read Archive are available at https://doi.org/10.17617/3.HVW8ZN.

## 1. Introduction

The vertebrate brain is one of the most functionally important and biologically complex structures of the body, making research on this organ of extreme interest yet difficult to study without supporting resources. By providing species specific information on the location and spatial relationships between anatomical and cytological features, brain atlases are an essential resource for neuroscience research (Hess et al., 2018; Arnatkevičiūtė et al., 2019). Many brain atlases have been created and applied to rodents (Yamamoto et al., 2001; Radtke-Schuller et al., 2016; Ortiz et al., 2020; La Manno et al., 2021) and primates (Newman et al., 2009; Sunkin et al., 2013; Moirano et al., 2019; Agaronyan et al., 2022) to better understand neurological processes ranging from circadian rhythms to neurodegenerative disease. However, a direct focus on these few mammalian lineages misses many of the naturally occurring phenotypes unique to other species that may prove pivotal for understanding brain function and evolution. For example, brain atlases created for the mustached bat (Washington et al., 2018), mole-rat (Dollas et al., 2019), and cavefish (Jaggard et al., 2020) have helped elucidate the adaptive mechanisms of sensory systems in darker environments. Continued curation of brain atlases across divergent species with extraordinary phenotypes will help to further broaden our understanding of brain function, architecture, and evolution.

A unique yet understudied brain phenotype is the drastic seasonal and reversible brain size change known as Dehnel’s phenomenon (Dehnel, 1949) that occurs in a handful of small mammals with exceptionally high metabolic rates and year-round activity (LaPoint et al., 2016; Nováková et al., 2022). The common shrew, *Sorex araneus*, has one of the most dramatic size changes found to date, thus, it is currently being used as model species for Dehnel’s phenomenon (Dehnel, 1949; Lázaro and Dechmann, 2021). Young common shrews reach their first maximum brain size soon after birth in summer, followed by a progressive reduction in brain size, reaching a minimum in winter. Partial regrowth of their brains occurs in spring as they sexually mature, followed by reproduction at approximately 13 months, after which most die prior to a second winter (Churchfield, 1979). In Southern Germany, the mean relative brain size of common shrews decreases by 16.1% from summer to winter and later regrows by 9.8% (for detailed data on brain mass see Lázaro et al., 2019). Notably, brain regions do not change uniformly, as each brain region shows different seasonal variation (Lázaro et al., 2019), and size changes are not driven by adult neurogenesis, apoptosis (Bartkowska et al., 2008) or changes in neuron size (Lázaro et al., 2018). Therefore, the curation of a brain atlas for *S. araneus* can further resolve region-specific shrinkage and regrowth at a finer resolution and help elucidate the molecular underpinnings of this rare phenotype.

Here we present two brain atlases as well as a region-specific gene expression profile to facilitate research on the common shrew. Our first atlas is a traditional histological atlas compiled from histological sections paired with schematic drawings in which the major brain regions and structures have been identified. Although histological atlases are useful resources for easy comparisons between taxa, they provide only bidimensional data and can cause tissue distortions or other artifacts that can slightly alter the shape of the brain structures (Ullmann et al., 2015). Thus, we also compiled a second, three-dimensional atlas using Magnetic Resonance Imaging (MRI), a powerful tool for obtaining detailed anatomical information at a finer resolution (Ullmann et al., 2015). Finally, we characterized the RNA expression profiles of 5 major brain regions; the cortex, hippocampus, hypothalamus, thalamus, and olfactory bulb. Measurements of gene abundance identified 1,444 genes of high regional specificity and large expression divergence between tissues. With these three datasets, we aim to create a resource for the scientific community to study this fascinating phenomenon with many potential applied questions.

## 2. Materials and methods

### 2.1. Histological sections

We prepared histological sections at the Max Planck Institute of Animal Behavior in Möggingen, Germany. We caught animals in the area near the institute between August 2013 and October 2015 (see Lázaro et al., 2018 for details of capture). Before brain extraction, animals were perfused transcardially with PBS followed by 4% formaldehyde solution in PBS under deep anesthesia (Isoflurane). We used the left hemisphere of 10 individuals (5 men and 5 women). Brain tissues were sectioned on a freezing sliding microtome (Reichert- Jung Hn-40) to obtain 30 μm-thick coronal sections, mounted every fifth section on slides, and stained them with 0.5% cresyl violet (see Lázaro et al., 2018 for details about sampling and preparation of sections). For the atlas, we then selected the best sections from the 10 individuals’ left hemisphere. To outline all brain regions, we used an Olympus BX51 microscope under an Olympus UIS2 Plan N 2 × (NA = 0.02) dry objective, inter-faced with a Neurolucida software system (MBF Bioscience, Williston, VT, USA). We then identified brain regions based on the cytoarchitecture revealed by this stain and used the mouse brain atlas as reference (Allen Mouse Brain Atlas; Paxinos and Franklin, 2019). The full list of brain regions identified can be found in Supplementary Table 1.

### 2.2. MRI data acquisition

For magnetic resonance imaging (MRI) reconstruction, we used one adult male common shrew. The individual was euthanized using deep isoflurane overdose and perfused through the open heart with phosphate-buffered saline (PBS) (see Lázaro et al., 2018 for details). The head was then removed and stored in PBS/0.1% sodium azide at 4°C. We performed MRI data acquisition with the brain preserved inside the skull to avoid tissue distortion and damage (Ullmann et al., 2015). Imaging was performed at the Universitätsklinikum Freiburg, Germany, using a BioSpec70/20 system (Bruker Biospin, Ettlingen, Germany) equipped with a BGA12S gradient insert with a cryogenically cooled 2-channel Tx/Rx mouse head surface coil. After tuning and matching the two coil channels’ standard adjustments and an oblique single-slice pilot scan, we performed a multi-slice pilot centered within the brain. Field homogeneity was optimized via *mapshim* defining the shim volume of an ellipsoid containing the complete brain.

For morphological imaging, a T2-weighted three-dimensional Rapid Imaging with Refocused Echoes (RARE) sequence with a turbo factor of eight and an isotropic resolution of 100 μm was employed. With a TEeff of 40 ms and a TR of 3,000 ms, a matrix of 320 × 160 × 120 at a field of view of 16 mm × 16 mm × 12 mm was acquired within 2 h.

To support the delineation of brain region boundaries a segmented, spin echo, 3D Diffusion Tensor Imaging sequence with an acquisition time of 12 h and 4 min was used. It used 10 segments, a TE/TR of 44/1,000 ms, 40 diffusion directions, and achieved a resolution of 100 μm isotropic with a field of view of 28.8 mm × 14 mm × 10.6 mm and a data matrix of 288 × 140 × 106.

### 2.3. MRI data post-processing

We analyzed the RARE images with the Nora Medical Imaging Platform (Anastasopoulos et al., 2017)^1^, a software for medical image processing developed by Universitätsklinikum Freiburg. We manually segmented brain regions of interest. The brain regions analyzed were *olfactory bulb*, *neocortex*, *caudoputamen*, *nucleus accumbens*, *amygdala*, *hippocampus*, *thalamus*, *hypothalamus*, *medulla*, *midbrain*, *pons*, and *cerebellum*. We used the histological atlas of the common shrew as well as the mouse brain atlas to identify brain regions (Allen Mouse Brain Atlas; Paxinos and Franklin, 2019). When in doubt about the identity of a particular structure due to image quality, no identification was made.

The diffusion weighted images were first denoised by a post-processing technique which uses random matrix theory (Veraart et al., 2016). This was followed by Gibbs artifact removal based on local sub-voxel shift (Kellner et al., 2016) and finally up-sampled to isotropic resolution by an edge-preserving interpolation approach (Kellner et al., 2016).

### 2.4. RNA extraction, library preparation, and sequencing

We extracted RNA from five individuals caught in November, 2019 from five different brain regions: *neocortex*, *hippocampus*, *hypothalamus*, *thalamus*, *and olfactory bulb*. A five individual sample size was chosen *a priori* to maximize the power of our differential expression analyses, while also not depleting our sampling population (Todd et al., 2016). We used a modified Qiagen Micro RNA easy protocol for RNA extractions that previous research in our lab (Yohe et al., 2020) has created specifically for small amounts of mammalian neuro-sensory tissue described below. We ground tissues using glass mortar and pestles on dry ice for 1–2 min to limit the degradation of RNA through temperature increases. Carrier RNA (5 ml of 4 ng/ml) and dithiothreitol (DTT, 7 ml at 2 M) is added to the 350 ml of lysate to improve lysing and binding. This mixture is added to each sample while in the glass mortar and ground for an additional minute. Following disruption with mortar and pestle, each reaction was further homogenized with QIAshredder columns. After homogenization, we followed the standard Qiagen Micro RNAeasy protocol, with a slight reduction in DNAse time (from a 15- to a 2-minute incubation), which we have found sufficient to reduce DNA contamination while minimizing RNA degradation. RNA extracted was sent to Azenta Life Sciences for quality control, library preparation, and sequencing. Azenta Life Sciences measured RNA quantity with a nanodrop and quality with RNA ScreenTape. RNA quality is measured with RNA Integrity Numbers (RINs), which quantifies RNA degradation by calculating RNA fragmentation. Generally speaking, RINs between 8 and 10 are high-quality samples, while those ranging from 6 to 8 are partially fragmented. RNA libraries were prepared with standard PolyA selection and sequenced with attempted depth of 15–25 million reads per sample using 150 bp paired-end reads.

### 2.5. Differential gene expression analysis

Adapters were trimmed and reads filtered using fastp (Chen et al., 2018). Filtered reads were quantified by pseudoaligning to the *S. araneus* genome (sorAra2; GCF_000181275.1) using Kallisto (Bray et al., 2016). Read counts were then normalized using the median of ratios in DESeq2 (Love et al., 2014). This normalization accounts for the library size of each sample and gene content. We conducted principal component analysis (PCA) of our samples using the top 500 varying genes across all our samples and plotted the principal components that explained the most variance. Using the distance between samples on PC space, we then hierarchically clustered the gene expression profiles and visualized the clustering on a heatmap of z-scores of the count data. We tested for differential expression in all five brain regions using DESeq2 by comparing the expression of genes in each region against all other brain regions at the same time. This was done to avoid multiple pairwise comparisons of each region against all other regions individually, as well as to identify region-specific genes. *P*-values were corrected for multiple testing with the Benjamini and Hochberg (1995) procedure. Significant differentially expressed genes were then filtered for those with an 1.58 log-fold change (absolute threefold change), to identify differentially expressed genes of high effect in the data set. Thresholds higher than this threshold are exceedingly rare in the brain, with minimal improvement in power (Todd et al., 2016). Finally, as differential gene expression analyses were largely exploratory, no *a priori* hypotheses were defined.

## 3. Results

We displayed the brain atlas of the common shrew as a series of histological sections and MRI images. We visualized landmarks in a series of sections and compared their position between images to match the alignment of MRI images to the histology sections. We identified 24 landmarks in the cerebrum, 19 in the brain stem, six in the cerebral nuclei, and three in the retrohippocampal region (Supplementary Table 1). The complete set of histological sections is available online at https://doi.org/10.17617/3.HVW8ZN.

**FIGURE 1:**
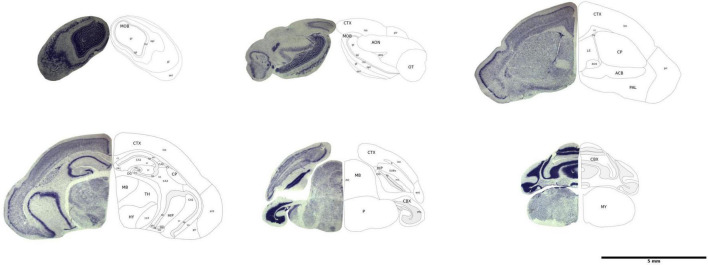
Representative histological brain sections with outlines of the brain regions. The image has been modified from Lázaro et al. (2018).

**FIGURE 2:**
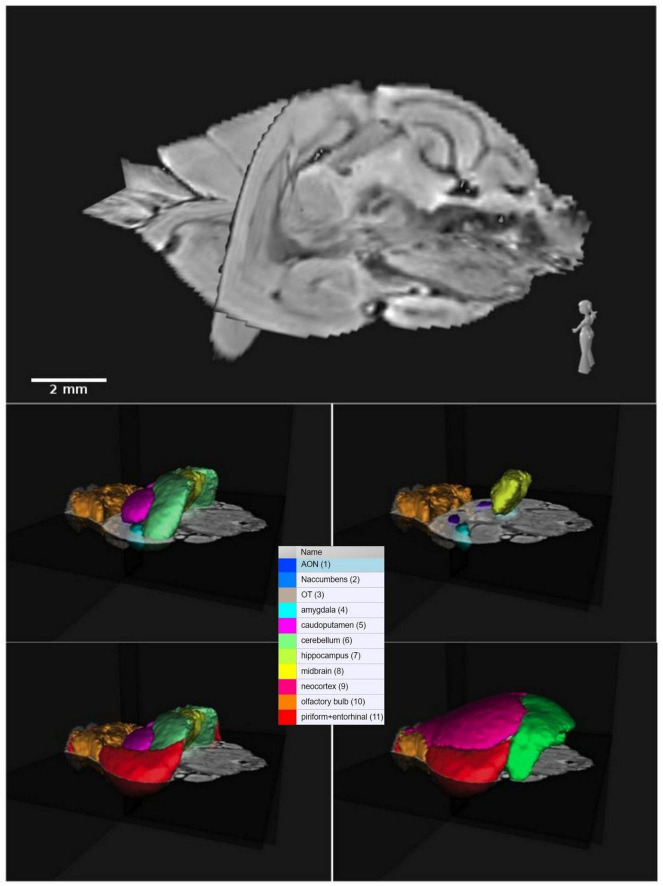
Three-dimensional orthogonal representation of the common shrew brain and related color-labeled brain regions. The acronyms correspond to: AON, anterior olfactory nuclei; OT, olfactory tubercle; Naccumbens, nucleus accumbens.

We identified 15 of the histological section using magnetic resonance imaging (Supplementary Table 1). A three-dimensional reconstruction of the common shrew brain can help understand relationships that are lost in two-dimensional sections (see representative sections in Figure 1; 3D orthogonal reconstruction of the brain in Figure 2) thus, the three-dimensional brain atlas based on MRI data is made available online on https://doi.org/10.17617/3.HVW8ZN. MRI data are provided in NIFTI format, which can be uploaded to the Nora Medical Imaging Platform. Users can browse and visualize the atlas as well as the delineations of brain regions using the open online interface (see text footnote 1).

### 3.1. RNA sequencing

RNA was extracted from five individuals for five brain regions: neocortex, hippocampus, hypothalamus, thalamus, and olfactory bulb (Supplementary Table 1). Extraction of a single individual hypothalamus produced no RNA, with no remaining tissue for further extraction, and this is not included in these results. Total sample reads ranged from approximately 15–27 million reads. RNA integrity Numbers (RIN) varied slightly between different brain regions; neocortex (6.4–8.6, mean 7.5), olfactory bulb (6.5–7.6, mean 7.1), hippocampus (7.0–8.6, mean 7.9), thalamus (5.6–7.8, mean 6.6), and hypothalamus (5.7–8.7, mean 7.1). We mapped reads to the reference transcriptome using Kallisto, with mapping rates ranging from 42.3–56.5%. This range excludes a hypothalamus sample with a mapping rate of 13.9%, however, we did not remove this sample from the experiment, as we normalized by library size prior to examining differential expression. The full list of samples with RIN and Accession Numbers can be found at https://doi.org/10.17617/3.HVW8ZN.

### 3.2. Transcriptomics

**FIGURE 3:**
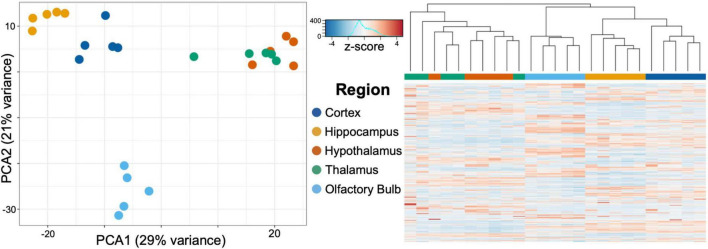
Principal component analysis (PCA) of the 750 most varying genes across brain regions characterized through RNA-seq. PC1 accounted for 35% and PC2 for 22% of the variance in gene expression. PC1 separated the olfactory bulb, hippocampus, and cortex from both the thalamus and hypothalamus. PC2 accounted for the variance from the olfactory bulb and the remaining brain regions. Hierarchical clustering of the samples confirms the unique expression profiles of the cortex, hippocampus, and olfactory bulb, while the hypothalamus and thalamus are largely indistinguishable from each other with these samples.

After normalizing using the median of ratios used in DESeq2, we transformed expression counts into log scale, and ran a principal component analysis (PCA) on the 750 genes in our data set with the most variance (Figure 3). The highest two principal components accounted for 35% (PC1) and 22% (PC2) of the variance found in the gene expression between regions. PC1 largely distinguished the olfactory bulb, hippocampus, and neocortex from the thalamus and hypothalamus, while PC2 accounted for the variance between the olfactory bulb and the rest of the brain regions. We then hierarchically clustered the samples using Euclidean distance between samples on principal component space (PC1 and PC2) (Figure 3). The neocortex, hippocampus, and olfactory bulb cluster into individual brain regions, while the hypothalamus and thalamus cluster together and could not be distinguished using these data.

**FIGURE 4:**
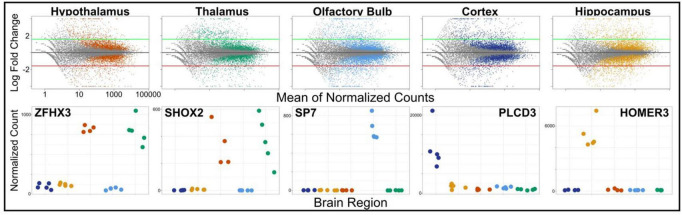
Gene expression data for each brain region (hypothalamus, thalamus, olfactory bulb, cortex, and hippocampus) plotted as the mean of normalized counts for each tissue over the log fold-change between tissue. Significant differentially expressed genes for each tissue are colored (*p* < 0.05). Green and red thresholds (±1.58 log fold-change) show differential expression of high effect. Examples of differentially expressed genes (ZFHX3, SHOX2, SP7, PLCD3, and HOMER3) are plotted below.

**FIGURE 5:**
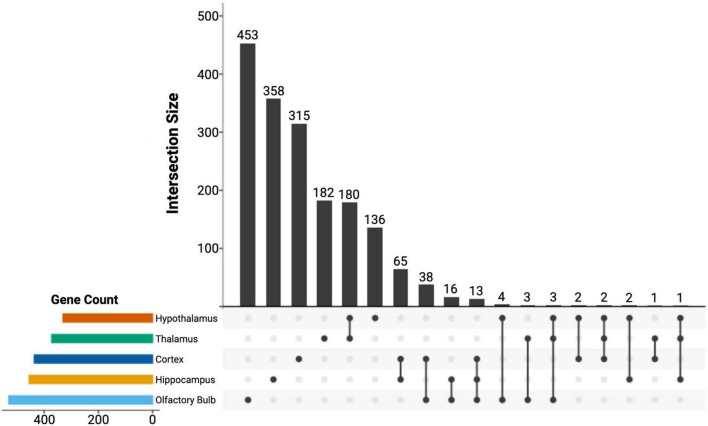
UPSET plot of the significantly differentially expressed genes for all the sampled brain regions. The set size measured the number of differentially expressed genes for each region, while the interaction size quantified the overlap between the below regions. A total of 1,444 differentially expressed genes do not have any overlap between brain regions. We also identified an overlap of 180 genes between the hypothalamus and thalamus, which further suggested the similarity of gene expression in these two regions.

Next, we used DESeq2 to test for differential expression between brain regions and set a log-fold change threshold of 1.58 (absolute fold change = 3), to determine how many of the differentially expressed genes were of high effect (Figure 4). The neocortex had 4,619 differentially expressed genes (2,505 upregulated and 2,115 downregulated) in respect to the other brain regions. Of these, 436 were highly upregulated, and 245 highly downregulated. We found similar numbers of differentially expressed genes in the hippocampus, with 4,380 differentially expressed genes (2,220 upregulated and 2,160 downregulated), of which 455 had high upregulation and 390 had high downregulation. The olfactory bulb had more differentially expressed genes (5,100) than both the hippocampus and neocortex, which validates its divergence in PCA. Of these, 2,468 were upregulated in comparison to the other tissues, with 530 highly upregulated, while 2,632 were downregulated, with 507 highly downregulated. The hypothalamus had fewer differentially expressed genes compared to other tissues (3,450; 1,864 upregulated and 1,586 downregulated), as well as less highly differentially expressed genes (330 upregulated and 241 down regulated). This pattern continued into the thalamus, with 3,527 differentially expressed genes (1,739 upregulated and 1,788 downregulated), and a few differentially expressed genes at a high level, 372 upregulated and 242 downregulated. This pattern is caused by the highly similar expression profiles of the thalamus and hypothalamus and is evident from the shared significance in each tissue in *ZFHX3* and *SHOX2* genes (Figure 5). We identified which genes were significant and of high effect in multiple tissues (Figure 5) and found an overlap of 180 genes in both the hypothalamus and thalamus, found in no other pair of brain regions, further suggesting that even differentially expressed genes in these two regions have a very similar expression.

## 4. Discussion

We created the first high-resolution brain atlas for the common shrew, *S. araneus* as a resource for neuroanatomical guidance of the common shrew brain. We developed two morphometric brain atlases using histological and MRI-based approaches to facilitate fundamental and applied research on the dramatic reversible brain size changes in individual common shrews. First, we generated a histological atlas from juvenile shrews. Here we identified a total of 52 brain structures throughout the cerebrum, brain stem, cerebral nuclei, and retrohippocampal region (Supplementary Table 1). At this stage of shrew development, the brain is at its largest but beginning to decrease in size. When looking at the brains of shrews from other ages researchers can expect brain regions to differ in size (see Lázaro et al., 2018 for details). Second, we visualized and identified regions of an old adult with MRI imaging (intermediate-sized brain compared to summer juveniles and winter subadults). Of the 52 brain structures identified in our histological analysis, we validated 15 with MRI imaging. Incomplete overlap between the two atlases occurs as some structures are more difficult to delineate on the MRI images while others are only partially available in the histological sections. The combination of the two atlases will allow researchers with different infrastructure to access information about the common shrew’s brain. While easily interpretable histological sections will be accessible to many collections, MRI data will allow researchers to visualize distinct neuroanatomical structures in three-dimensional space. Although MRI yields a reduction in the number of recognizable nuclei due to resolution limitations, the ability to locate brain structures using the accurate coordinate system is an advantage of this method. By combining traditional morphological (histology), advanced imaging (MRI), and RNA expression (transcriptomics), this new atlas can guide diverse future studies with a range of technologies.

We also produced and integrated transcriptomic data, further validating brain regions based on expression profiles. While each of our focal brain regions in this method (cortex, hippocampus, hypothalamus, thalamus, and olfactory bulb) had expression profiles consisting of thousands of differentially expressed genes (Figure 4), we identified 1,444 non-overlapping, highly differentially expressed genes across regions (Figure 5). Some of these genes include ZFHX3 (hypothalamus), SHOX2 (thalamus), SP7 (olfactory bulb), PLCD3 (cortex), and HOMER3 (hippocampus) (Figure 4). By comparing these genes to results to data found in the Human Protein Atlas,^2^ we found that while SHOX2 tissue specificity matches human, mouse, and pig thalamus specificity, the remaining genes are not brain-region-specific in humans but are in either pig or mouse. Although brain regions between species have similar expression profiles, differences in brain expression have been reported before (Pucek, 1963). Therefore, the divergence in region specificity between species found here is not uncharacteristic of mammalian brain expression. Our findings validate the need for our species-specific transcriptomic atlas for *S. araneus* to determine the molecular mechanisms of Dehnel’s phenomenon, while also identifying a need for further characterization and analysis of evolutionary change in brain expression.

By focusing on the species that is most frequently studied for the brain size changes occurring during Dehnel’s phenomenon, we intend to contribute to improved knowledge of the mammalian brain. Despite the long history of research on common shrew biology, the dramatic changes happening in the brain remain almost entirely unexplained. Variations in water and lipid content contribute significantly to the seasonal size fluctuations but do not explain them completely (Pucek, 1963), and the proximal causes of the morphological changes at the cellular and molecular levels remain unknown (Bartkowska et al., 2008; Lázaro et al., 2018).

This study focuses on generating a reference atlas of the common shrew brain, rather than quantifying the seasonal change in volume. As a result, we choose not to display the three age groups in the atlas and instead concentrate on developing a useful template using representative data. Moreover, although the changes can be quite significant based on the brain region, they have no impact on the structure of the brain as a whole. Thus, future research endeavors must tackle these unanswered questions through repeated *in vivo* MRI to study the different stages of brain size in the same individuals or using Diffusion Tensor Imaging (DTI) which would allow non-invasive tracking of brain white matter fibers, enabling researchers to determine how water travels differently across seasons. Furthermore, region-specific genes identified here can be used to validate regions as they are analyzed through shrew brain development. This atlas will both improve the common shrew as a model for future neuroscientific studies, and help understand the processes that contribute to brain regeneration in mammals, with potential implications for the biology of human neurodegenerative diseases.

## Data availability statement

The data presented in this study are deposited in the Edmond repository, https://doi.org/10.17617/3.HVW8ZN.

## Ethics statement

This animal study was reviewed and approved by the all experimental procedures were carried out according to guidelines for the care and use of animals approved by the Regierungspräsidium Freiburg, Baden-Württemberg (35-9185.81/G-11/21 and 35-9185.81/G-19/162).

## Author contributions

CB, DD, MM, and JL captured the shrews and extracted the brains. CB, JL, and MM prepared the histological sections and the histology atlas. LD supervised. WT performed the gene expression analyses. MM, MR, DE, and CB designed the 3D atlas. CB, WT, DE, DD, LD, and JN wrote the manuscript. CB and WT prepared the figures. All authors discussed the results and contributed to the final version of the manuscript.

## Footnotes

1
https://www.nora-imaging.org

2
https://www.proteinatlas.org/ENSG00000161714-PLCD3/brain
