# Supplementary material for: Histological and MRI brain atlas of the common shrew, Sorex araneus, with brain region-specific gene expression profiles
Source: Front Neuroanat. 2023 May 3;17:1168523. doi: 10.3389/fnana.2023.1168523 (PMC10188933; doi:10.3389/fnana.2023.1168523)
Supplement: Supplementary file 1 [file Table_1.DOCX]

Supplementary Material

**Histological and MRI brain atlas of the common shrew, *Sorex araneus*, with brain region specific gene expression profiles**

**Cecilia Baldoni^*^†, William Thomas†, Dominik von Elverfeldt, Marco Reisert, Javier Làzaro, Marion Muturi, Liliana M. Davalos, John D. Nieland, Dina K. N. Dechmann**

*** Correspondence:** Cecilia Baldoni cbaldoni@ab.mpg.de

# Supplementary **Table**

**Table 1. List of brain structures identified in histological sections and MRI, with corresponding abbreviations. In the MRI atlas, Piriform and entorhinal areas are grouped together.**

|  | **Histology** | **MRI** | **Gene Expression** |
| --- | --- | --- | --- |
| Cerebrum: |  |  |  |
| CTX | cerebral cortex | X | X |
| MOB | main olfactory bulb | X | X |
| mi | mitral layer |  |  |
| gr | granule layer |  |  |
| opl | outer plexiform layer |  |  |
| ipl | inner plexiform layer |  |  |
| gl | glomerular layer |  |  |
| onl | olfactory nerve layer of main olfactory bulb |  |  |
| AON | Anterior olfactory nucleus |  |  |
| OT | olfactory tubercle | X |  |
| pir | piriform area | X |  |
| aco | anterior commissure, olfactory limb |  |  |
| iso | isocortex |  |  |
| HIP | hippocampal region | X | X |
| CA1 | CA1 field |  |  |
| CA2 | CA2 field |  |  |
| CA3 | CA3 field |  |  |
| sr | stratum radiatum |  |  |
| so | stratum oriens |  |  |
| sp | pyramidal layer |  |  |
| slm | stratum lacunosum- moleculare |  |  |
| DG | dentate gyrus |  |  |
| mo | dentate gyrus, molecular layer |  |  |
| FA | fasciola cinerea |  |  |
| Retrohippocampal region: |  |  |  |
| ent | entorhinal area | X |  |
| SUBd | Subiculum, dentral part |  |  |
| SUBv | Subiculum, ventral part |  |  |
| Cerebral Nuclei: |  |  |  |
| STR | Striatum |  |  |
| ACB | Nucleus accumbens | X |  |
| CP | caudoputamen | X |  |
| LS | Lateral septal nucleus |  |  |
| sAMY | Striatum- like amygdalar nuclei | X |  |
| PAL | pallidum |  |  |
| Brain stem: |  |  |  |
| HY | hypothalamus | X | X |
| TH | thalamus | X | X |
| MB | midbrain | X |  |
| P | pons | X |  |
| MY | medulla | X |  |
| CBX | cerebellum | X |  |
| PFL | paraflocculus |  |  |
| Fi | fiber tracts: |  |  |
| cc | corpus callosum |  |  |
| VL | lateral ventricle |  |  |
| V3 | third ventricle |  |  |
| AQ | cerebral aqueduct |  |  |
| aco | anterior commissure, olfactory limb |  |  |
| act | anterior commissure, temporal limb |  |  |
| Cpd | cerebral peduncle |  |  |
| sg | granule cell layer |  |  |
| po | polymorph layer |  |  |
| SUBd | Subiculum, dentral part |  |  |
| SUBv | Subiculum, ventral part |  |  |
